# A Novel Deep Neural Network Technique for Drug–Target Interaction

**DOI:** 10.3390/pharmaceutics14030625

**Published:** 2022-03-11

**Authors:** Jackson G. de Souza, Marcelo A. C. Fernandes, Raquel de Melo Barbosa

**Affiliations:** 1 Laboratory of Machine Learning and Intelligent Instrumentation, Federal University of Rio Grande do Norte, Natal 59078-970, Brazil; jackson.souza@gmail.com; 2 Department of Computer Engineering and Automation, Federal University of Rio Grande do Norte, Natal 59078-970, Brazil; 3 Department of Pharmacy and Pharmaceutical Technology, Faculty of Pharmacy, University of Granada, 18071 Granada, Spain

**Keywords:** drug–target interaction, DTI prediction, deep learning, convolutional neural network

## Abstract

Drug discovery (DD) is a time-consuming and expensive process. Thus, the industry employs strategies such as drug repositioning and drug repurposing, which allows the application of already approved drugs to treat a different disease, as occurred in the first months of 2020, during the COVID-19 pandemic. The prediction of drug–target interactions is an essential part of the DD process because it can accelerate it and reduce the required costs. DTI prediction performed *in silico* have used approaches based on molecular docking simulations, including similarity-based and network- and graph-based ones. This paper presents MPS2IT-DTI, a DTI prediction model obtained from research conducted in the following steps: the definition of a new method for encoding molecule and protein sequences onto images; the definition of a deep-learning approach based on a convolutional neural network in order to create a new method for DTI prediction. Training results conducted with the Davis and KIBA datasets show that MPS2IT-DTI is viable compared to other state-of-the-art (SOTA) approaches in terms of performance and complexity of the neural network model. With the Davis dataset, we obtained 0.876 for the concordance index and 0.276 for the MSE; with the KIBA dataset, we obtained 0.836 and 0.226 for the concordance index and the MSE, respectively. Moreover, the MPS2IT-DTI model represents molecule and protein sequences as images, instead of treating them as an NLP task, and as such, does not employ an embedding layer, which is present in other models.

## 1. Introduction

Drug discovery (DD) is a time-consuming and expensive process in which, despite the recent technological advancements and the increasing investments, most of the compounds examined fail during clinical trials or due to toxic and adverse side effects [1,2,3]. Furthermore, existing drugs may become less effective due to drug resistance [4].

The industry aims to find new uses for already approved drugs, avoiding the expensive and lengthy process of drug development [5,6]. This strategy is known as: (i) drug repositioning, which usually refers to the studies that reinvestigate existing drugs that failed approval for new therapeutic indications [7], and (ii) drug repurposing, which suggests the application of already approved drugs and compounds to treat a different disease [8,9]. For instance, in the first months of 2020, approximately 70 existing FDA-approved drugs were under investigation to see if they could be re-purposed to treat COVID-19 [7]; disease-modifying pharmacotherapies (such as nilotinib, inosine, and isradipine) are being repurposed to treat Parkinson’s Disease [10]; the repurposing in cardiovascular diseases of drugs approved and marketed for other pathologies [11]; genome-wide association studies (GWASs) that involve the use of human genetic data to link genes to specific diseases have already resulted in candidate targets for drug discovery and repurposing [12]; and the drug Sildenafil, originaly developed for treating pulmonary hypertension, was repurposed as Viagra to treat erectile dysfunction in men [1].

Drug–target interaction (DTI) refers to the binding of a drug (a chemical compound) to a target (proteins or nucleic acids) that results in a change in its biological behavior/function, bringing it back to normal [1,13]. The prediction of DTI is an essential part of the DD process because it can accelerate it and reduce the required costs [14], but it is difficult and costly, as experimental assays not only take significant time but are expensive [15]. Therefore, researchers have intensified research into the identification of the relationship between drugs and targets, hoping to accelerate the pace of drug development and shorten the time to market [16].

DTI predictions performed on a computer (in silico) can be used to effectively estimate the interaction strength of new drug–target (DT) pairs based on previous DT experiments, accelerating the DD process by systematically suggesting a new set of candidate molecules, dealing with the high amount of complex information (e.g., hydrophobic interactions, ionic interactions, hydrogen bonding, and/or van der Waals forces) between molecules [15,17,18].

Four types of *in silico* DTI prediction methods have been proposed in the literature: molecular docking, similarity-based, deep-learning-based, and network-based models [15,19]. Molecular docking is a simulation-based method using the 3D-structured features of a molecule (ligand) and a protein (receptor) [15,20,21]. Similarity-based methods are based on the hypothesis that similar drugs share similar targets, and vice versa [19], and use two similarity matrices: (a) drug similarity (computed by chemical structures) and (b) target similarity (generated by protein sequence alignment) [22,23,24]. Network-based methods are derived from algorithms used in recommender systems and link prediction algorithms in complex networks [19]. This paper focuses on deep-learning approaches for DTI prediction, and some related works are presented below.

Deep-learning-based methods can steer experiments, reveal latent patterns in large-scale drug or protein data collections and extract unprecedented knowledge in drug–target networks [14]. Most approaches rely on features to calculate feature descriptors (fingerprints) for both drugs and targets, the DT pair, which is then used for training a classifier, resulting in the desired DTI prediction [13]. Some of these methods are: (i) stacked auto-encoders of deep learning [16]; (ii) convolution neural networks (CNN) [25]; (iii) molecular graph convolution (MGC) [26]; (iv) ensembles of multi-output bi-clustering trees (eBICT) [14]; (v) network-based inference (NBI) methods derived from recommendation algorithms [19]; (vi) the combination of multiple kernels into a tripartite heterogeneous drug–target–disease interaction spaces in order to integrate multiple sources of biological information simultaneously [3]; and (vii) graph neural network models [18]. Further information on feature-based and machine learning methods for DTI can be found in [1,4,13,22,27].

More recently, following the advancements of natural language processing (NLP), embedding techniques were used for pre-training and transfer-learning via fine-tunning. A modified BERT Transformer [28] was used for molecule input embedding [15], and a BERT Transformer was used to propose SMILES-BERT, a semi-supervised model for molecule embedding [29]. The word2vec model [30] was used to represent the embedding of genes [31]. Graph neural networks (GNNs) and Bi-LSTM [32] were used to propose a graph and sequence fusion learning model that captures significant information from both a SMILES sequence and a molecular graph [33]. Four text-based information sources, namely, the protein sequence, ligand SMILES, protein domains and motifs, and maximum common substructure words, were used to predict binding affinity [34].

Feature descriptors of molecules and proteins play an important role in the DTI and other genomics-related process because machine learning needs the inputs to be mapped to another representation, including: one-hot encoding [16,35,36]; graphs or networks [14,18,19]; character embedding [3,15,25]; extended connectivity fingerprints (ECFP) for molecule representation and protein sequence composition (PSC) for protein representation [26]; and a custom molecule representation in which each sequence symbol is considered as a time point [37]. The layers of a deep neural network, such as CNN, are arranged in order to define the model’s architecture that, applied to DTI task, automatically extract features from input data, represents knowledge, and provides the desired prediction [15,25].

This paper presents two main contributions: a new approach for mapping molecule and protein sequences to their respective image-based representations, named here as a molecule and protein sequence to image transformer (MPS2IT), and a CNN-based architecture which receives the molecule and protein image representations as inputs and outputs the prediction of their drug–target interaction. Therefore, by using these two elements, this research results in a new method for DTI prediction, called here a molecule and protein sequence to image transformer DTI (MPS2IT-DTI).

The experimental results presented in this paper show that, compared to the state-of-the-art (SOTA) approaches, our method provides a viable alternative to the NLP-based techniques, resulting in the analysis of images instead of text-based sequences of molecules and proteins. In order to provide this conclusion, the paper compares the results obtained with the MPS2IT-DTI model against KronRLS [23], Simboost [24], DeepDTA [25], WideDTA [34], and MT-DTI [15] approaches.

## 2. Molecule and Protein Sequence to Image Transformer DTI (MPS2IT-DTI)

This section presents a new drug–target interaction (DTI) model and a new mapping approach to represent molecule and protein sequences as images, i.e., matrices of numerical values. One motivation of this research is that current state-of-the-art methods based on deep-learning networks are addressing the DTI prediction task by focusing mainly on the architecture of the deep-learning model. Moreover, motivated by the research on the NLP task with deep learning, current approaches rely on complex text-learning models with transfer learning, which require massive computational power. On the other hand, the approach presented by this paper focuses on the mapping of the textual input to a numerical representation (which is a 2D image) and applying a deep-learning architecture based on a dual-CNN for molecule and protein input. Similar approaches have been adopted in the literature, as can be seen in [38].

**Figure 1:**
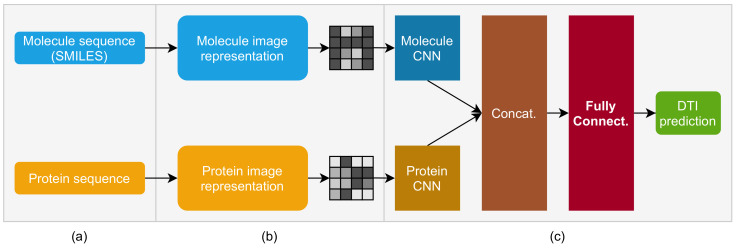
Molecule and protein sequence to image transformer DTI prediction model: (**a**) input: molecule sequence (SMILES) and protein sequence; (**b**) representation: molecule and protein sequences are mapped to image pixels; (**c**) CNN-based deep neural network for DTI prediction: molecule and protein input images are fed into molecule and protein CNNs, whose output is concatenated and fed into a dense, fully connected network, whose output is the DTI prediction.

The MPS2IT-DTI model, presented in this paper and illustrated in Figure 1, receives an input composed of two elements, a molecule sequence (representing the drug) and a protein sequence (representing the target), and then predicts their interaction value. However, in order for this model to operate, the inputs cannot be expressed as character sequences, because the CNN model, which is part of MPS2IT-DTI, needs numerical values as inputs [39].

The MPS2IT-DTI model receives two inputs (Figure 1a): the molecule sequence (its SMILES [40] representation) and the protein sequence (its sequence of amino acids), i.e., the inputs are textual character sequences.

The representation (Figure 1b) is a process that maps the textual input to an image (mathematically, a matrix of real numbers between 0 and 1). This mapping technique is based on *k*-mers counting [41,42,43] and feature frequency profiles (FFP) [44] to create an image that represents the unique signature of the sequence.

The deep neural network model (Figure 1c) has two branches, composed of two CNNs, one for the molecule and another for the protein. The two CNNs are concatenated to represent the input to a fully connected neural network. The output of the fully connected network represents the DTI prediction between the model’s input, i.e., the molecule sequence and the protein sequence. The following sections present each element of this model in detail, starting by the mapping of a molecule sequence to an image.

### 2.1. Mapping Molecule Sequence to an Image

**Figure 2:**
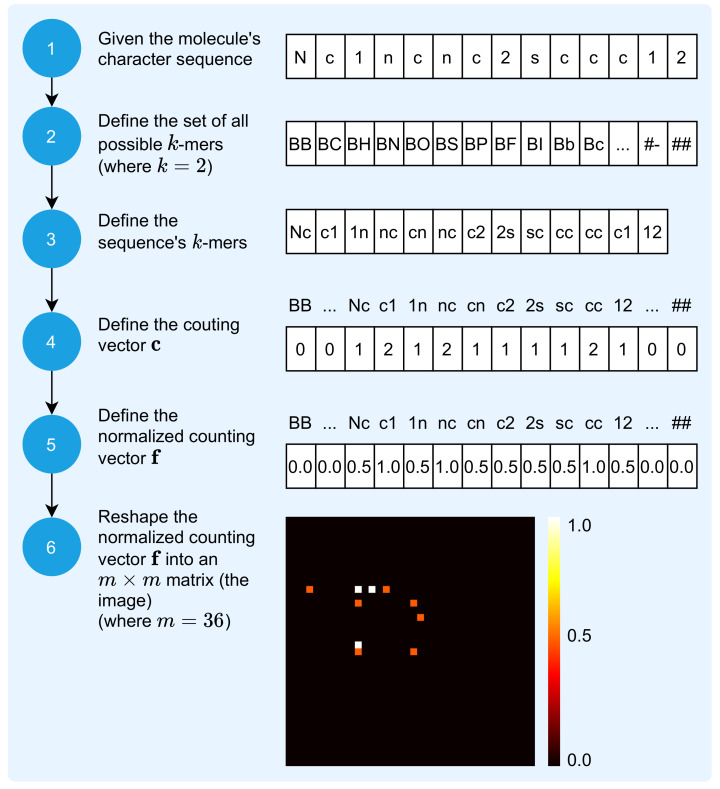
Molecule sequence to image transformation.

The mapping of the molecule sequence, i.e., the molecule SMILES representation, to an image is a process that receives the molecule sequence and outputs its visual unique signature. The 2D image that results from this mapping process is a matrix of numeric values within the closed interval (0,1). The process is composed of six steps, as depicted in Figure 2.

The characters, or symbols, of the SMILES 1D representation are elements of the set $\Sigma_{M}$, which has n=36 elements and is defined by:

(1) $$\Sigma_{M}=\left\{B,C,H,N,O,S,P,F,I,b,c,l,n,o,s,p,r,0,1,2,3,4,5,6,7,8,9,\left(,\right),\left[,\right],=,.,+,-,\#\right\}.$$

Each $\sigma \in \Sigma_{M}$ is used in the representation of the molecule’s sequence $s_{M}$, defined by:

(2) $$s_{M}=\left[s_{1},s_{2},\ldots ,s_{c}\right],$$

where *c* is the sequence length.

The first step is to represent the molecule as a sequence of characters. Figure 2 illustrates the mapping of the molecule with identification CHEMBL1972934 in PUBCHEM, also known as Thieno[2,3-d]pyrimidin-4-amine [41], defined by:

(3) $$s_{M}=\left[N,c,1,n,c,n,c,2,s,c,c,c,1,2\right].$$

Thus, this molecule is represented by a sequence comprised of c=14 characters.

The second step is to define the set of all possible *k*-mers, defined by:

(4) $$K=\{k_{1},k_{2},\ldots ,k_{n^{k}}\},$$

where $n^{k}$ is the amount of all possible *k*-mers and each $k_{i}\in K$ is the *i*-th *k*-mer, which is a sequence, or word, of *k* symbols, considering the set $\Sigma_{M}$. For k=2, there are $36^{2}=1296$ possible *k*-mers.

The third step is to define the vector $w_{M}$, the sequence’s *k*-mers, defined by:

(5) $$w_{M}=\left[w_{1},w_{2},\ldots ,w_{l}\right],$$

where each $w_{i}\in w_{M}$ is the *i*-th *k*-mer and l=c−k+1 defines the amount of the molecule sequence’s *k*-mers. For k=2 and the molecule illustrated by Figure 2, l=14−2+1=13.

The fourth step involves the definition of the counting vector c, defined by:

(6) $$c=[c_{1},c_{2},\ldots ,c_{n^{k}}],$$

where $c_{i}\in c$ is the number of occurrences of each $k_{i}\in K$ (see Equation (4)) in the molecule’s sequence $s_{M}$. The next step involves normalizing the counting vector c, starting with the definition of the normalization factor *f*, defined by:

(7) f=maxc,

and continuing, with the definition of the normalized counting vector f as:

(8) $$f=\frac{1}{f}\times c.$$

The sixth step results in the image representation of the molecule’s sequence, the matrix $I_{M}$, which is obtained by reshaping the normalized counting vector f into a squared m×m matrix, with *m* defined by:

(9) $$m=*\sqrt[{2}]{n^{k}}.$$

Each $I_{i,j}$ element, or pixel, is a real number in the closed interval (0,1) that represents the number of occurrences of each possible *k*-mer in the sequence. If $n^{k}\neq m^{2}$, then extra

(10) $$e=\left|m^{2}-n^{k}\right|$$

elements with value 0 are concatenated to the end of the normalized counting vector f, before the reshaping process, for additional padding.

**Figure 3:**
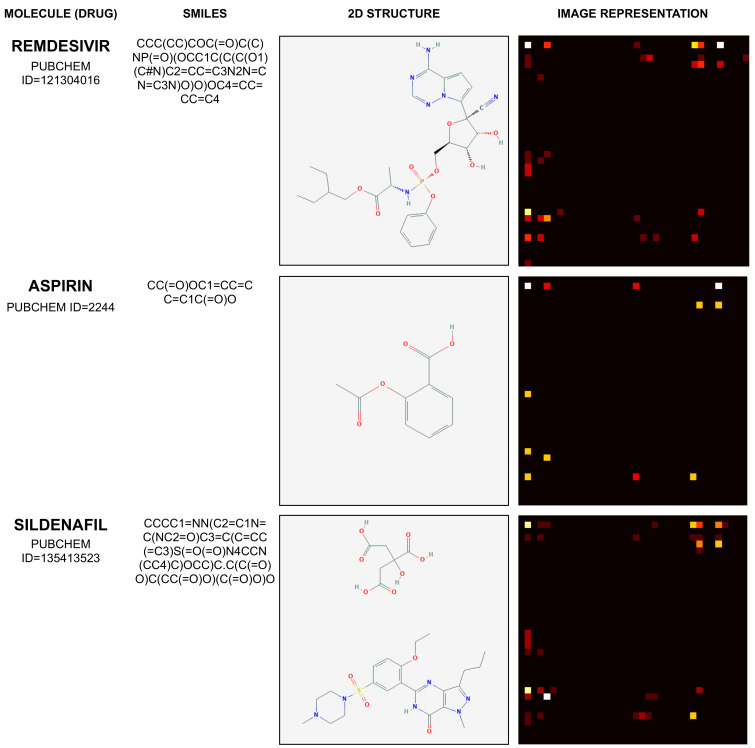
Example of mapping molecule sequence to image.

Therefore, the mapping process illustrated by Figure 2 results in an image with dimensions of 36×36, with pixel values between 0 and 1. Considering the molecules Remdesivir, Aspirin, and Sildenafil, Figure 3 illustrates their SMILES (1D) representation, chemical (2D) representation, and their related image representation as obtained from the method described above.

The molecules depicted by Figure 3 and their corresponding PUBCHEM identifiers are Remdesivir (121304016), Aspirin (2244), and Sildenafil (135413523). Their 1D SMILES representation and 2D visual structure representation are obtained from [41].

### 2.2. Mapping Protein Sequence to an Image

**Figure 4:**
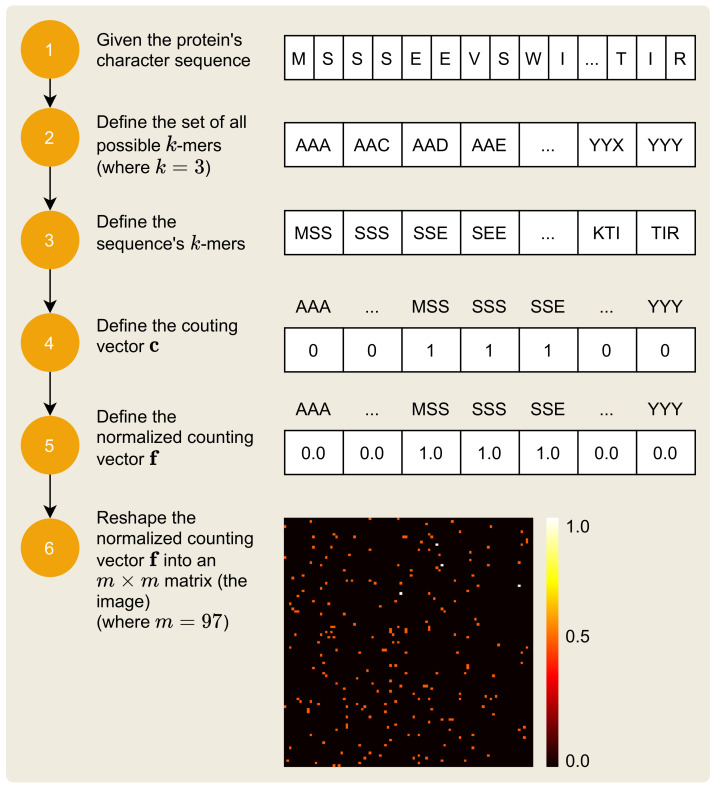
Protein sequence to image transformation.

The mapping of the protein sequence, i.e., the protein’s various amino acids, to an image is a process that receives the protein sequence and outputs its unique visual signature. The 2D image that results from this mapping process is a matrix of numerical values, within the closed interval (0,1). The process is composed of six steps, as depicted in Figure 4.

The symbols of the protein sequence can be represented by a sequence of the symbols of the set $\Sigma_{P}$, of size n=21, and which is defined by:

(11) $$\Sigma_{P}=\left\{A,C,D,E,F,G,H,I,K,L,M,N,P,Q,R,S,T,V,W,X,Y\right\}.$$

Each $\sigma \in \Sigma_{P}$ is used in the representation of the protein’s sequence $s_{P}$, defined by:

(12) $$s_{P}=\left[s_{1},s_{2},\ldots ,s_{c}\right],$$

where *c* is the sequence length.

The six-step process to represent a protein sequence as an image, illustrated by Figure 4, is similar to the process presented in Section 2.1, but there are some remarkable differences. First, the protein has identification P67870 in UNIPROT, also known as Casein kinase II subunit beta [45]. This protein is defined by the sequence:

(13) $$s_{P}=\left[M,S,S,S,E,E,V,S,W,I,\ldots ,T,I,R\right],$$

which has c=215 characters. Equation (4) remains the same, but with k=3 and $\Sigma_{P}$, there are $n^{k}=21^{3}=9261$ possible *k*-mers. Equation (5) also remains the same, but there are l=215−2+1=214*k*-mers in the protein sequence $s_{P}$. The next steps (4–6), involving the counting vector c, the normalized counting vector f, and the resulting image matrix $I_{P}$, remain the same with the difference in the dimension of the resulting image matrix: since m=97, the resulting image has dimensions of 97×97.

**Figure 5:**
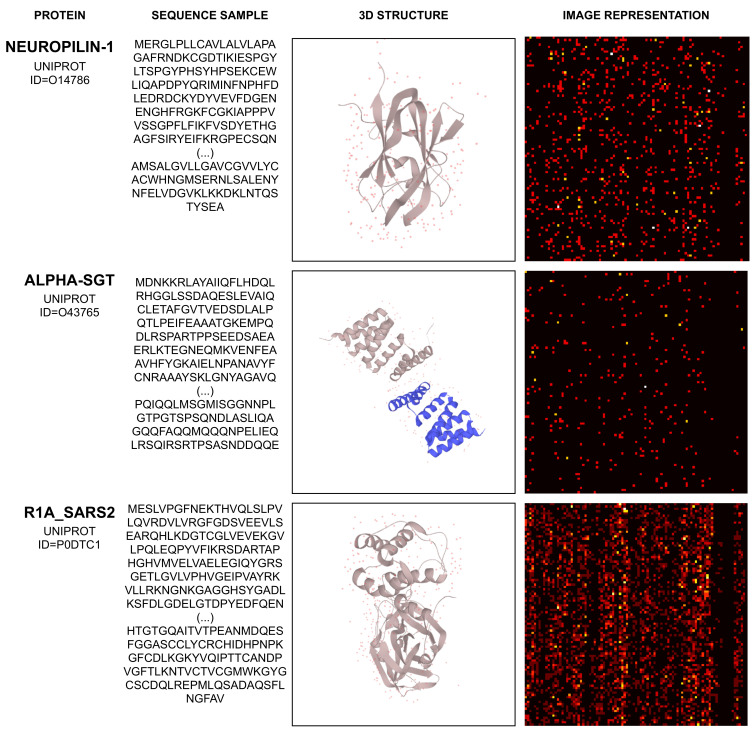
Example of mapping protein sequence to image.

Considering the proteins Neuropilin-1, Alpha-SGT, and R1A_SARS2, Figure 5 illustrates their sequence sample, chemical (3D) representation, and their related image representation, as obtained from the method described above.

The proteins depicted by Figure 5 and their corresponding UNIPROT IDs are Neuropilin-1 (O14786), Alpha-SGT (O43765), and R1_SARS2 (P0DTC1). Their amino acid sequence and 3D structure’s visual representation are obtained from [45].

This section presented the mapping technique which generates an *m*-by-*m* image from a molecule sequence and from a protein sequence, as they are necessary for the deep neural network of the DTI prediction model to operate. Section 2.3 continues to present the MPS2IT-DTI prediction model by the definition of the molecule and protein CNNs.

### 2.3. Molecule and Protein CNNs

**Figure 6:**
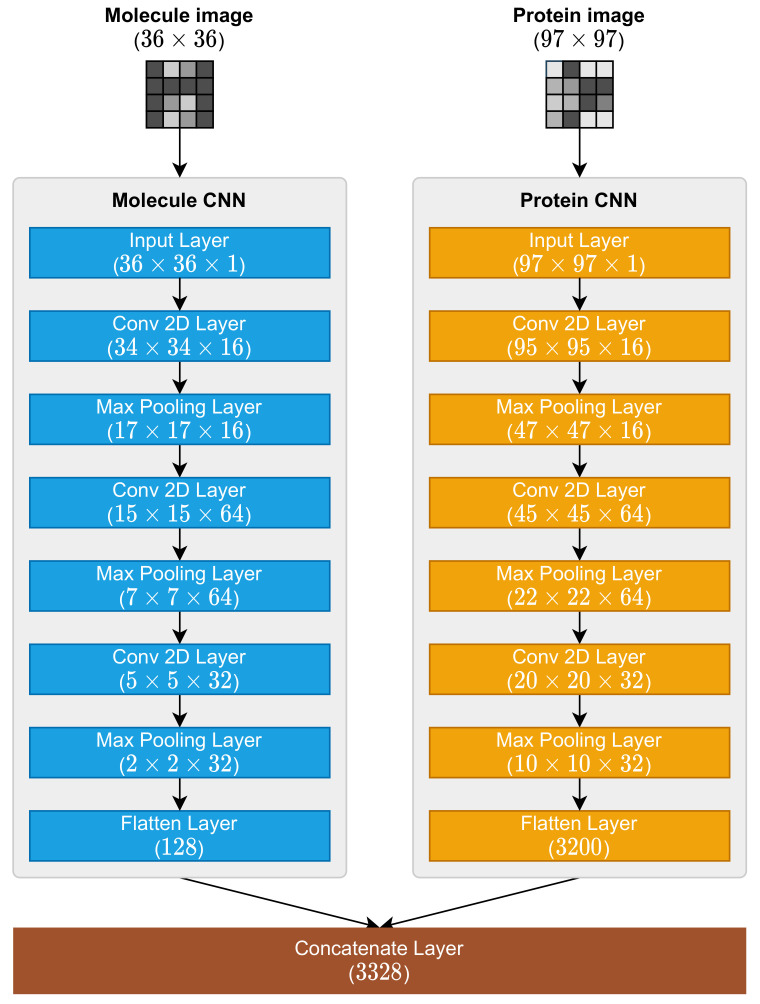
Molecule and protein CNNs, their layers, and the concatenate layer.

It is important to remember that the mapping process presented in Section 2.1 and Section 2.2 were responsible for the representation necessary for these CNNs to operate because the MPS2IT-DTI prediction models receive the molecule and protein character sequences (Figure 6). Because of this dual input, the MPS2IT-DTI model has two branches, each one dealing with molecule and protein inputs. Before using the fully connected network to predict the interaction between molecule and protein, the MPS2IT-DTI model uses two CNNs: one for the molecule image and another for the protein image.

The molecule and protein CNNs are responsible for feature extraction from their respective inputs. The molecule CNN extracts features from the molecule image representation, while the protein CNN extracts features from the protein image representation. These CNNs share a common structure, being composed by the following layers:

1. Input Layer;
2. Conv 2D Layer (with ReLu activation function);
3. Max Pooling 2D Layer;
4. Conv 2D Layer (with ReLu activation function);
5. Max Pooling 2D Layer;
6. Conv 2D Layer (with ReLu activation function);
7. Max Pooling 2D Layer;
8. Flatten Layer.

The shapes (dimensions) of each layer differs because of each input shape: $I_{M}$ is (36×36), and $I_{P}$ is (97×97). Figure 6 illustrates the architecture of molecule and protein CNNs.

These CNNs outputs are, then, concatenated by one concatenate layer whose output shape is (3328). This concatenate layer output is fed into a fully connected network, which is described in the next section.

### 2.4. Fully Connected and Output Blocks

The remaining structure of the proposed DTI model is composed of the following layers:

1. Dropout Layer (0.4 rate);
2. Dense Layer (dimension (512), with ReLu activation function);
3. Dropout Layer (0.4 rate);
4. Dense Layer (dimension (1), with linear activation function).

**Figure 7:**
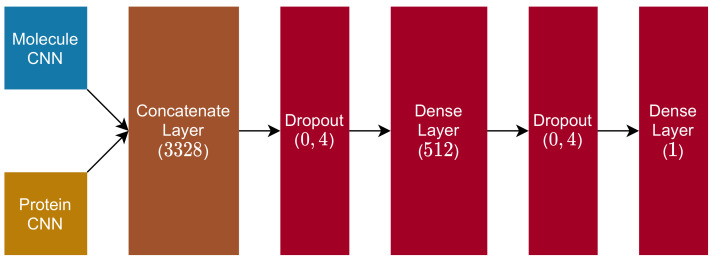
Fully connected and output blocks.

Figure 7 illustrates this composition and provides a notion about how this fully connected block is positioned in the MPS2IT-DTI model as it receives the concatenate layer output.

After the molecule CNN and the protein CNN are concatenated into one vector of dimension 3328, the model applies a dropout layer, is then followed by a dense layer (with ReLu activation), another dropout (with the same rate as the previous one), and a final dense layer (with linear activation), whose output is the DTI prediction between the molecule and protein fed into the model.

## 3. Materials and Methods

This research results in a new mapping technique, used to represent molecule and protein sequences as images, and a new CNN-based DTI prediction model, MPS2IT-DTI, which receives molecule and protein sequences as inputs and results in the DTI prediction. This section presents the datasets, the molecules and proteins to image transformations, the metrics, the baselines, as well as detailed information about the model training, validation, and testing.

### 3.1. Datasets

The MPS2IT-DTI model is evaluated on two benchmarks: Davis [46] and KIBA [47]. They have been used for evaluation in previous DTI studies [15,23,24,25], whose results were used as baselines for comparison. Davis is comprised of large-scale biochemical selectivity assays for clinically relevant kinase inhibitors with their respective dissociation constant ($K_{d}$) values, while KIBA combines three scores $K_{i}$, $K_{d}$, and $IC_{50}$ by optimizing consistency among them [15]. As suggested by [24], the $K_{d}$ values are transformed into the log space, defining $pK_{d}$ as:

(14) $$pK_{d}=-\log_{10}\left(\frac{K_{d}}{1e9}\right).$$

The original KIBA dataset represents 246,088 affinity bindings interactions among 467 proteins and 52,498 molecules (drugs). Ref. [24] filtered the dataset by removing all the proteins and molecules with <10 interactions, resulting in the dataset used in this research. Table 1 summarizes the number of drugs, targets (proteins), and interactions of the two datasets.

**Figure 8:**
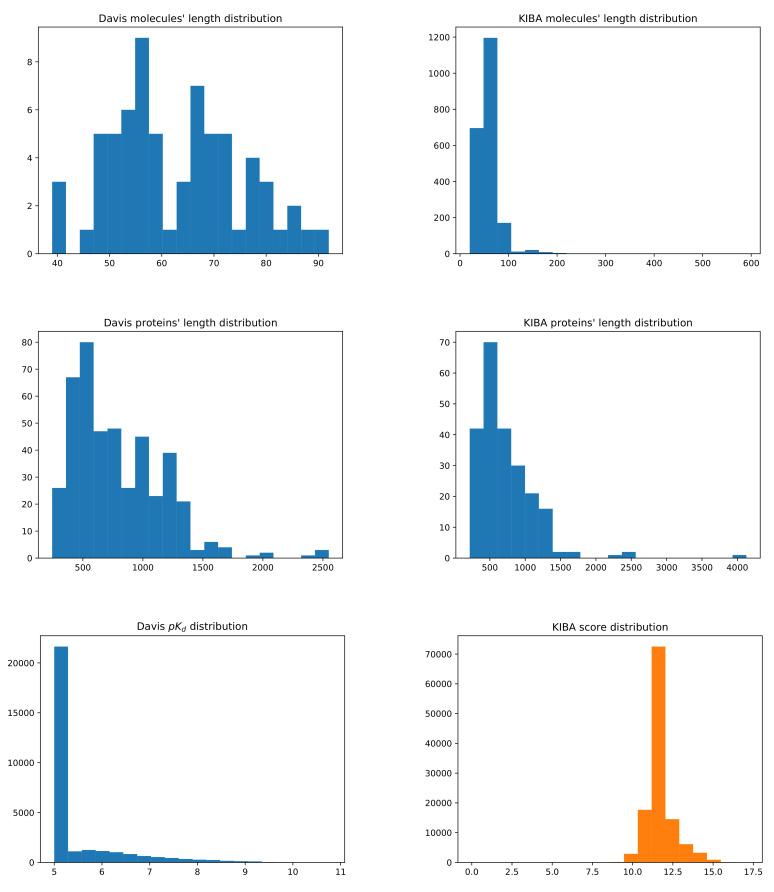
Summary of the Davis (**left**) and KIBA (**right**) datasets. The first row shows the distribution of the molecules’ length representation in SMILES. The second row shows the distribution of the proteins’ sequence length. The third row shows the distribution of binding affinity values, respectively, $pK_{d}$ and KIBA score.

Furthermore, Figure 8 depicts the structure of the Davis and KIBA datasets by provinding the histrograms of the distributions of molecules’ and proteins’ lengths, along with $pK_{d}$ and KIBA score values.

The Davis dataset contains molecules whose SMILES representation length is, at minimum, 39 at maximum 103, and at average 64, while the proteins’ sequences’ minimum length is 244, maximum is 2549, and average is 788. For the KIBA dataset, the mininum, maximum, and average molecules’ SMILES lengths are 20, 590, and 59, while for proteins they are 215, 4128, and 728, respectively. Lower values for $K_{d}$ and KIBA scores represent better binding affinities. However, for Davis, because of $pK_{d}$ definition in Equation (14), higher values represent better affinity binding.

The molecules’ and proteins’ sequences present in the Davis and KIBA datasets were transformed according to the molecule and protein sequence to image representation presented in Section 2.1 and Section 2.2. For the molecules, the *k*-mers value was set to 2 and, for proteins, the *k*-mers value was set to 3. This resulted in molecule images with 36×36 pixels and protein images with 97×97 pixels. This process did not interfere with the number of drugs, targets, or interactions presented earlier.

### 3.2. Evaluation Metrics

Four evaluation metrics were used to evaluate MPS2IT-DTI model: mean squared error (MSE), concordance index (CI) [48], $r_{m}^{2}$, and area under the precision–recall curve (AUC-PR). MSE is defined as the average of the sum of the squared differences between true $\hat{y_{i}}$ and predicted $\hat{y_{i}}$ labels:

(15) $$\mathrm{MSE}=\frac{1}{n}\sum_{i=1}^{n}{\left(\hat{y_{i}}-y_{i}\right)}^{2},$$

where i∈(1,n) and smaller values indicate better prediction performance. The CI is the probability that the predicted scores of two randomly chosen drug–target pairs, $y_{i}$ and $y_{j}$, are in the correct order if their corresponding true affinity scores, $\hat{y_{i}}$ and $\hat{y_{j}}$, satisfy $\hat{y_{i}}>\hat{y_{j}}$ [15]:

(16) $$\mathrm{CI}=\frac{1}{N}\sum_{\hat{y_{i}}>\hat{y_{j}}}h\left(y_{i}-y_{j}\right),$$

where *N* is a normalization constant that represents the number of pairs in the correct order and h(x) is a step function defined as [25]:

(17) $$h\left(x\right)=\left\{\begin{array}{cc} 1, & \;\mathrm{if}\;x>0\\ 0.5, & \;\mathrm{if}\;x=0\\ 0, & \;\mathrm{else} \end{array}\right.$$

The $r_{m}^{2}$ index is defined as [15]:

(18) $$r_{m}^{2}=r^{2}\left(1-\sqrt{r^{2}-r_{0}^{2}}\right),$$

where $r_{2}$ and $r_{0}^{2}$ are, respectively, the squared correlation coefficients with and without intercept defined as:

(19) $$r^{2}=\frac{{\left(\sum \left(Y_{pred}-\bar{Y_{pred}}\right)\left(Y_{obs}-\bar{Y_{obs}}\right)\right)}^{2}}{\sum {\left(Y_{obs}-\bar{Y_{obs}}\right)}^{2}\times \sum {\left(Y_{pred}-\bar{Y_{pred}}\right)}^{2}},$$

(20) $$r_{0}^{2}=1-\frac{\sum {\left(Y_{obs}-k\times Y_{pred}\right)}^{2}}{\sum {\left(Y_{obs}-\bar{Y_{obs}}\right)}^{2}},\;\mathrm{and}$$

(21) $$k=\frac{\sum (Y_{obs}\times Y_{pred})}{\sum {\left(Y_{pred}\right)}^{2}},$$

where $Y_{obs}$ and $Y_{pred}$ are the observed and predicted values and $\bar{Y_{obs}}$ and $\bar{Y_{pred}}$ are the mean observed and predicted values, respectively. An acceptable model has $r_{m}^{2}>0.5$ [15].

The area under the precision–recall curve (AUC-PR) is a model performance metric for binary responses that is appropriate for rare events and not dependent on model [49,50]. For the AUC-PR metric, as it is used for binary classification, the regression scores for Davis ($pK_{d}$) and KIBA were transformed into the binary labels 1 (binding) and 0 (no binding). This approach was also used by previous researchers [15,24,47]:

(22) $$pK_{d}=\left\{\begin{array}{c} 1,\;\mathrm{if}\geq 7\\ 0,\;\mathrm{else} \end{array}\right.\;\mathrm{KIBA}\mathrm{score}=\left\{\begin{array}{c} 1,\;\mathrm{if}<12.1\\ 0,\;\mathrm{else} \end{array}\right.$$

This methodology allowed for an understanding of how MPS2IT-DTI performed in relation to the baseline approaches, as is presented next.

### 3.3. Baselines

Five baseline methods were used to compare the MPS2IT-DTI performance: KronRLS [23], Simboost [24], DeepDTA [25], MT-DTI [15], and WideDTA [34]. KronRLS is a similarity-based model whose goal is to minimize the MSE loss function with a regularization term given as a norm of the prediction model [23]. Simboost is another similarity-based method which is based on a gradient-boosting machine and utilizes specific metrics such as networks metrics and latent vectors from matrix factorization [24].

While KronRLS and Simboost employ traditional machine learning methods, DeepDTA, MT-DTI, and WideDTA are deep-learning-based models. DeepDTA employed one-hot encoding to transform the SMILES representation of the molecules’ and the proteins’ sequences into two embedding layers, generating a two-input model where two CNNs were used to extract features from the inputs. MT-DTI evolved from DeepDTA by improving the molecule representation based on the self-attention mechanism. WideDTA, also based on DeepDTA, employed an approach that explored the combination of four different sources of textual information and, instead of using a character-based model (as in DeepDTA), it used words as input.

### 3.4. Training Details

**Figure 9:**
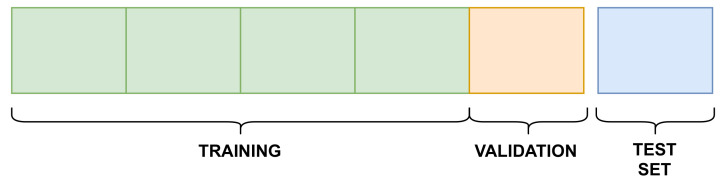
Experiment setup.

The training of the MPS2IT-DTI model used the Davis and KIBA datasets. In order to provide comparison and reproducibility, the same 5-fold cross validation setup with a held-out test set provided by [25] and that is publicly available (https://github.com/hkmztrk/DeepDTA/, accessed on 7 February 2022) were used. Each dataset was divided into six equal parts: five for the 5-fold cross validation and one as an independent test set, as illustrated by Figure 9.

Figure 9 illustrates that, for each *k*-fold, four splits are used for training and one for validation. Table 2 presents the sizes of the training, validation, and testing splits for each dataset.

The same approach was used in all of the baseline methods (Section 3.3) and also in the experiments involved in the formulation of the MPS2IT-DTI model.

The main objective of the training is to minimize the MSE value. For such a task, the MPS2IT-DTI model used the Adam optimizer [51] with a learning rate of 0.001, β1=0.9, β2=0.999, and $\epsilon =10^{-7}$. The batch size was set to 256 and the training occurred for 1000 epochs.

For each *k*-fold, the training process was executed in a two-phase setting:

- Training–validation phase: this phase used the training and validation splits, which resulted in a trained model, stored to be used in the next phase;
- Testing phase: the trained model was tested against the testing split, generating the results for each evaluation metric.

Thus, this process resulted in 5 models, one for each *k*-fold. Their scores for each of the evaluation metrics were averaged in order to determine the model’s performance, which is presented next.

The MPS2IT-DTI model was implemented in the Python programming language and used the Tensorflow [52] and Keras [53] packages in order to run in a GPU (graphics processing unit) and accelerate the training process.

## 4. Results and Discussions

As introduced in Section 3.2, the drug–target binding affinity was modeled as a regression (prediction) and as a binary classification problem, according to the evaluation metrics used. The metrics CI (Equation (16)), MSE (Equation (15)), and $r_{m}^{2}$ (Equation (18)) were used, considering the regression problem. When the drug–target binding affinity was modeled as a binary classification problem, the AUC-PR metric was used to evaluate the model’s performance. Additionally, the Accuracy score (ACC) was also employed to understand how good was the model at considering a binary classification problem.

The training–validation–testing process presented in Section 3.4 allowed for a determination of the model’s performance, according to the evaluation metrics. The results are summarized in Table 3.

Table 3 allows us to note that the model’s general performance was better on Davis than on KIBA: +5% on CI, +22% on MSE, +4% on $r_{m}^{2}$, and +13% on accuracy. On AUC-PR, performance was +25% better on the KIBA dataset.

The model performance evaluation was compared to baseline reference methods (Section 3.3). Table 4 shows the results of MPS2IT-DTI related to the previous approaches.

Considering the Davis dataset, the model performance evaluation shows that the MPS2IT-DTI model performed similarly to the baselines according to the CI and MSE evaluation metrics: it performed higher than SimBoost (+0.46%) and lower than DeepDTA (−0.23%). For the $r_{m}^{2}$ evaluation metric, it performed better than DeepDTA (+2.17%) and worse than MT-DTI. For the AUC-PR metric, the model performed worse than all baselines.

Considering the KIBA dataset, the MPS2IT-DTI model performance was also very similar, considering the CI and MSE evaluation metric: higher than KronRLS (+6.46%) and lower than DeepDTA (−3.23%)—the performance was the same as SimBoost. For the $r_{m}^{2}$ evaluation metric, it performed higher than KronRLS (44.30%) and lower than SimBoost (−2.44%). For the AUC-PR metric, the model performed better for KIBA in relation to Davis, but also inferior to the baseline methods.

Furthermore, the MPS2IT-DTI model architecture was compared to DeepDTA, WideDTA, and MT-DTI, considering the model’s architecture in relation to their input, representation, and deep neural network. As seen in Section 2, all models employ a similar architecture composed of: input, representation, and a fully connected block, resulting in the DTI prediction. The MPS2IT-DTI model differs from the baseline methods, mainly considering how it deals with the input (Table 5) and representation (Table 6).

Table 5 shows that MPS2IT-DTI, DeepDTA, and MT-DTI use two inputs, while WideDTA adopts four inputs: two for representing the molecule (SMILES and ligand maximum common substructure) and two for representing the protein (protein sequence and domain/motif information). WideDTA explored the effect of additional pieces of specific information, trying to achieve a better modeling of the interactions [34].

Table 6 shows that, about the approaches adopted to create a molecule representation and a protein representation:

- MPS2IT-DTI employs an approach that represents molecule and protein sequences as two images that are fed into two CNNs;
- DeepDTA and WideDTA employ a similar approach, representing molecule and protein sequences as two embedding layers that are fed into two CNNs [25,34];
- MT-DTI employs a different approach for molecule and protein sequences: the molecule representation is based on the BERT model, with a multi-layer transformation; the protein representation follows the same approach as did DeepDTA and WideDTA [15].

Furhtermore, it is noticeable that even with the understanding that current SOTA deep-learning methods, especially those applied to NLP tasks, could not achieve known results without using the embeddings model, this does not come without the price of computational cost, which is to be considered, mainly in the sustainable technology context [54,55].

After the molecule and protein representations, all methods under analysis employ a concatenate layer, in order to create a single input vector which is fed into a fully connected network, which is composed of dense layers. Finally, all models’ outputs are similar, as they are employed to the same prediction task.

This section presented the performance of MPS2IT-DTI when applied as a prediction and as a binary classification model, considering the Davis and KIBA datasets. An analysis of the obtained performance shows that the performance was comparable to the baseline methods.

## 5. Conclusions

This research proposed a new deep-learning-based approach to predict drug–target binding affinity using sequences of drugs (molecules) and proteins. The first contribution was a new approach to represent molecules’ sequences and proteins’ sequences as images, i.e., a visual signature of molecules and proteins. Compared to the considered state-of-the-art (SOTA) approaches, this approach provides an alternative to the very well-known one-hot encoding, and results in a approach with lower complexity than the ones based on NLP techniques, which employ an embedding layer. The resulting deep-learning model, MPS2IT-DTI, uses images to represent and extract features from molecules’ sequences and proteins’ sequences and does not use an embedding layer, which also reduces the complexity of the model’s architecture.

This MPS2IT-DTI model shares elements found in the SOTA approaches, but because of the molecules’ and proteins’ sequences’ representation, the current approach was performed on images, operating mostly similarly to a CNN model that predicted the binding affinity between two images that represented their correspondent molecules and proteins.

The experiment setup employed in the learning phase of the baseline methods was also used in the MPS2IT-DTI model, showing that the approach was reproducible and comparable.

The analysis of the models’ performance showed that the model performed better than machine learning-based approaches and, specially considering the CI evaluation metric, it performed similarly to the other methods, but employed a less-complex approach.

## Figures and Tables

**Table 1:** Davis and Kiba datasets’ characteristics.

Dataset	No. Drugs	No. Targets	No. Interactions
Davis	68	442	30,056
Kiba	2111	229	118,254

**Table 2:** Training, Validation and Testing splits.

Dataset	Training	Validation	Testing
Davis	20,037	5009	5010
KIBA	78,836	19,709	19,709

**Table 3:** Detailed evaluation metric scores for each fold in the 5-fold cross validation.

Dataset	CI (std)	MSE	$r_{m}^{2}$ (std)	AUC-PR (std)	ACC (std)
Davis	0.876 (0.002)	0.276	0.637 (0.011)	0.450 (0.029)	0.944 (0.002)
KIBA	0.836 (0.003)	0.226	0.614 (0.011)	0.601 (0.006)	0.881 (0.002)

**Table 4:** Training and test results of the proposed MPS2IT-DTI model and other existing approaches.

Dataset	Method	CI (std)	MSE	$r_{m}^{2}$ (std)	AUC-PR (std)
Davis	KronRLS	0.871 (0.001)	0.379	0.407 (0.005)	0.661 (0.010)
	SimBoost	0.872 (0.002)	0.282	0.644 (0.006)	0.709 (0.008)
	**MPS2IT-DTI**	0.876 (0.002)	0.276	0.637 (0.011)	0.450 (0.018)
	DeepDTA	0.878 (0.004)	0.261	0.630 (0.017)	0.714 (0.010)
	WideDTA	0.886 (0.003)	0.262	–	–
	MT-DTI	0.887 (0.003)	0.245	0.665 (0.014)	0.730 (0.014)
Kiba	KronRLS	0.782 (0.001)	0.411	0.342 (0.001)	0.635 (0.004)
	**MPS2IT-DTI**	0.836 (0.003)	0.226	0.614 (0.011)	0.601 (0.006)
	SimBoost	0.836 (0.001)	0.222	0.629 (0.007)	0.760 (0.003)
	DeepDTA	0.863 (0.002)	0.194	0.673 (0.009)	0.788 (0.004)
	WideDTA	0.875 (0.001)	0.179	–	–
	MT-DTI	0.882 (0.001)	0.152	0.738 (0.006)	0.837 (0.003)

**Table 5:** MPS2IT-DTI model compared to baselines according to the inputs.

Inputs	MPS2IT-DTI	DeepDTA	MT-DTI	WideDTA
Num. of inputs	2	2	2	4
Molecule SMILES	•	•	•	•
Ligand Max. Common Structure	◦	◦	◦	•
Protein Sequence	•	•	•	•
Protein Motifs and Domains	◦	◦	◦	•

**Table 6:** MPS2IT-DTI model compared to baselines according to the representation of the inputs: (M) molecules and (P) proteins.

Approach	MPS2IT-DTI		DeepDTA		MT-DTI		WideDTA	
	M	P	M	P	M	P	M	P
Mers-based frequency	•	•	◦	◦	◦	◦	◦	◦
Frequency to image	•	•	◦	◦	◦	◦	◦	◦
One-hot encoding	◦	◦	•	•	◦	•	•	•
Embedding layer	◦	◦	•	•	•	•	•	•
Self-attention layer	◦	◦	◦	◦	•	◦	◦	◦
Feed-forward layer	◦	◦	◦	◦	•	◦	◦	◦
Pre-training	◦	◦	◦	◦	•	◦	◦	◦
Fine tunning	◦	◦	◦	◦	•	◦	◦	◦
CNN	•	•	•	•	◦	•	•	•
